# Prognostic factors in clear cell sarcoma: an analysis of soft tissue sarcoma in 43 cases

**DOI:** 10.1007/s00432-024-05980-3

**Published:** 2024-11-13

**Authors:** Janik Grothues, Jendrik Hardes, Abbas Agaimy, Stephane Collaud, Lars Podleska, Farhad Farzalyev, Nina Myline Engel, Rainer Hamacher, Benjamin Fletcher, Christoph Pöttgen, Stefanie Bertram, Hans-Ulrich Schildhaus, Arne Streitbürger, Sebastian Bauer, Johanna Falkenhorst

**Affiliations:** 1 Department of Musculoskeletal Oncology, Sarcoma Center, 45147 Essen, Germany; 2 Institute of Pathology, Friedrich-Alexander-University Erlangen-Nürnberg, University Hospital, Erlangen, Germany; 3 Lung Clinic, Department of Thoracic Surgery, Cologne Merheim Hospital, University of Witten/Herdecke, Witten, Germany; 4 Department of Medical Oncology and Sarcoma Center, West German Cancer Center, Medical School, University Duisburg-Essen, Essen, Germany; 5 Department of Radiotherapy, West German Cancer Centre, University Hospital Essen, 45147 Essen, Germany; 6 Institute of Pathology, University Medical Center Essen, 45147 Essen, Germany; 7 Discovery Life Sciences Biomarker GmbH and Pathologie Nordhessen, Germaniastr. 7, 34119 Kassel, Germany; 8 German Cancer Consortium (DKTK), Partner Site University Hospital Essen, Essen, Germany

**Keywords:** Clear cell sarcoma, CCS, Prognostic factors, Malignant melanoma of soft tissue, Gastrointestinal sarcoma, EWSR1

## Abstract

**Purpose:**

Clear cell sarcoma (CCS) of tendons and aponeuroses and CCS-like malignant gastrointestinal neuroectodermal tumor/sarcoma (GINET) are characterized by frequent local and distant relapses, alongside with low efficacy of all systemic treatments. We aimed to collect a comprehensive dataset to identify prognostic factors and treatment outcomes.

**Methods:**

We performed a retrospective single center analysis for diagnosed CCS and GINET on demographic, tumor, treatment and survival data.

**Results:**

We identified 43 patients (w:25, m:18) with a median follow-up of 35mo and a 5y-OS-rate of 42%. At diagnosis the median age was 42yrs. Median tumor size was 3.6 cm (0.3–11.1 cm), and 24/26 (94%) tissues analyzed at our institute were *EWSR1::ATF1*-translocation-positive. Distant extremities (incl. knee or elbow) were affected in 72.5%. Of note, 79.5% received an excisional biopsy (benign histology suspected in 30.2%) leading to frequent incomplete resection. Final R0 status correlated significantly (p = 0.017) with longer survival rates compared to R + status in localized CCS (N0M0, 5-yr OS 0% vs 64%). Radiation and systemic treatment had limited antitumor effects while isolated limb perfusion was active in some patients. 18.6% of patients showed lymphatic spread and 20.9% distant metastases. Presence of initial M + was associated with a dismal survival of 1.4 years (M +) vs 7.1 years (M0; p < .001).

**Conclusion:**

We here present one of the largest clinical cohorts of patients with CCS/GINET. Our data underscores the exceptional risk of metastatic disease even in small tumors. As systemic treatment and radiation showed limited efficacy, complete resection was the most important treatment option.

**Supplementary Information:**

The online version contains supplementary material available at 10.1007/s00432-024-05980-3.

## Introduction

Clear cell sarcoma (CCS) of tendons and aponeuroses was described in 1965 by Dr. Franz Enzinger (1965) as a rare sarcoma displaying melanocytic phenotype and representing ≤ 1% of all soft tissue sarcomas. Clinical presentation is a small tumor of the soft tissue primarily on the extremities (Enzinger 1965; Chung and Enzinger 1983). CCS are characterized by an aggressive clinical course with early-onset lymph node and distant metastases and a dismal long-term prognosis. Due to histological and immune-phenotypic similarities with melanomas, CCS have in the past been considered as ‘malignant melanoma of the soft parts’ but on clinical, anatomical and, as later became available, molecular grounds CCS are distinct. While CCS may contain melanin and it expressed regularly melanoma-associated antigens (HMB-45, SOX10, S-100 and others) (Chung and Enzinger 1983), the pathognomonic oncogenic event is a chimeric *EWSR1::ATF1* translocation, t(12;22) (q13;q12) (Zucman J et al. 1993). Mutations in the *BRAF* gene as well as a high mutational burden frequently found in melanoma are absent in CCS. GINET represent a CCS-like sarcoma with similar pathological appearance as well as a recurrent *EWSR1*-fusions, typically with a *CREB1* fusion mate t(12;22) (q34;q12) (Stockman et al. 2012; Green et al. 2018). However, GINET definitionally expresses S100/SOX10, but lacks specific melanocytic antigens (HMB45, MelanA, tyrosinase) and, instead, shows variable neuroectodermal markers such as synaptophysin, CD56 and others.

Few patient cohorts have been published and current staging systems fail to assist in determining the prognosis of patients. We aimed to analyze a large single-center cohort of patients to describe the clinical course, benefit to medical treatment and outcome of multimodal approaches.

## Material and methods

We identified 43 patients (n = 3 GINET) in the institutional database of the West German Cancer Center who have been treated or consulted between 1992 and 2022 with a median follow-up of 35 months. Only cases that united clinical characteristics, histopathological, immunochemical and particularly genetic features typical for CCS/GINET were included in this study and those not distinguishable from melanoma were excluded, based on the following criteria: superficial localization with infiltration of the epidermis vs. deep location with relation to tendons and aponeuroses in CCS, high number vs. low number of mitoses in CSS, frequent BRAF mutation and absent EWSR1-fusion (Anna M. Czarnecka et al. 2024). Histopathological criteria for CCS that were used to confirm diagnosis was the presence of round to fusiform cells arranged in solid nests or fascicles with clear to eosinophilic cytoplasm (Enzinger 1965; Deenik et al. 1999) forming a neuroendocrine-like organoid growth pattern (Kosemehmetoglu and Folpe 2010). Immuno-histological staining for melanocytic differentiation included Melan-A, HMB-45 and S-100 in most (Chung and Enzinger 1983; Kosemehmetoglu and Folpe 2010; Deroose et al. 2011; Deenik et al. 1999) as well as a few patients with Vimentin, SOX-10 (Stockman et al. 2012) and bcl-2 (Kosemehmetoglu and Folpe 2010; Hisaoka M et al. 2008) markers. *EWSR-ATF1/-CREB1* fusion was detected by fluorescence in situ hybridization (FISH) assay in Formalin-fixed paraffin-embedded tissues and in fresh frozen tumor tissue transcriptase-polymerase chain reaction (RT-PCR) was the method of choice. All tumor samples from patients included in this report were reviewed by reference pathologies for sarcoma and the diagnosis ‘clear cell sarcoma’ or ‘GINET’ was established.

Covariates included gender, age, location, depth, symptoms, size (≤ 5 or > 5 cm), clinical staging, resection status, treatment with isolated limb perfusion (ILP), radiotherapy (RTx), and chemotherapy (CTx). Clinical staging has been updated to 8th edition of Union of International Cancer Control and American Joint Committee on Cancer (UICC/AJCC) guidelines for soft tissue sarcomas (Amin et al. 2017). For the TNM stadium T1 is defined as ≤ 5 cm, T2 ≤ 10 cm, T3 ≤ 15 cm. No T4 (> 15 cm) tumors were found in this cohort. For validating current staging systems, we categorized CCS as high-grade tumors, given the well documented, poor outcome of patients diagnosed with this disease. Thus, stage I (T1-T4, N0, M0, G1) was not represented. The term *‘localized’* disease was used for N0 and M0 status. *Locoregional spread* included positive lymph node involvement (N1) as well as the presence of satellite (< 2 cm of primary) or in-transit metastases and skip metastasis. Patients with the diagnosis of metastases within three months of the diagnosis of the primary tumor were categorized as ‘synchronous metastases’ and those later than three months represented ‘metachronous metastases’. *Recurrence* subsumes tumor reappearance after local resection, novel lymph nodes or distant metastasis at least three months after complete resection of the primary (Gaakeer H.A. et al. 1988).The tumor depth was measured retrospectively in relation to the fascia, or to tendons and aponeuroses using preoperative CT/MRI scans.

Treatments applied to patients included surgery, chemotherapy (CTx), radiotherapy (RTx) and isolated limb perfusion (ILP). Resection status was defined as R0: no residual tumor, R1: microscopic residual tumor and R2: macroscopic residual tumor. A ‘whoops’ procedure was defined as resection of a mass that is assumed to be benign at time of surgery but turned out to be a sarcoma upon pathological examination with positive surgical margins (R1 or R2). Duration of treatment as well as time to progression was determined using patient charts and radiology reports from each patient.

Statistical data for survival was generated using SPSS. GINET were statistically treated as CCS. All time-to-event endpoints were computed by using the Kaplan–Meier method. The log-rank test was used to compare survival differences between binary variables. A multivariate Cox regression was performed for survival analysis of the following time-independent covariates: age, gender, tumor location, locoregional lymph/distant metastasis at diagnosis and final resection status. In consistency with pre-existing retrospective analyses in CCS (Montgomery et al. 1993; Kawai et al. 2007; Hocar et al. 2012), p-values < 0.05 were considered significant because of the exploratory character in a relatively unexplored field. Patients’ follow-up ranged from 39 days to 26.9 years (median 35mos). In cases where the data were unavailable, they were excluded from analysis. This was a single-center retrospective cohort study. The study was conducted in accordance with the 1964 Helsinki Declaration and approved by the Ethics committee of University Duisburg-Essen (16–6757-BO).

## Results

### Clinical characteristics

The study consisted of 25 women (58.1%) and 18 men (41.9%) with CCS or GINET. The youngest patient was 12 years and the oldest 74 years of age. The median age at time of diagnosis was 41.5 years. Most patients presented with a palpable, asymptomatic mass at first diagnosis. Only few patients had additional symptoms with local pain (n = 9), swelling (n = 7), the appearance of a small and painless, movement related irritating knot close to tendons and aponeuroses (n = 4) or ulceration (n = 3). Frequently (n = 13) CCS were misinterpreted as benign tumors with ganglion (n = 5) representing the most frequent alternate diagnosis, followed by cyst (n = 3), bursitis (n = 3), blue nevus (n = 1) and hematoma (n = 1). The time from first symptoms to active treatment was 5.1 months (range: 1-33mos) but this information was available for six patients only.

The CCS primary tumors were mainly located within the lower extremity in 27 cases (62.8%) with the foot being most common (n = 12, 27.9%) followed by the knee area with 18.6% (n = 8). The hand was affected in four cases (9.3%). CCS typically arose in peripheral extremities (elbow or knee region and distal) in 72.5%. Only five cases originated from the body core (abdominal cavity). In one unusual case CCS was diagnosed in the cervix uteri after a Wertheim-Meigs surgery was performed. All GINET were located within the intestinal organs (n = 3).

Tumor size at time of diagnosis was 3.6 cm (median; range 0.3—11.1 cm) with most tumors (73.6%) being under 5 cm (n = 28). Synchronous metastatic disease (N + and/or M +  = stage IV AJCC) was observed in 39.5% (n = 17) whereas 18.6% showed only lymph node metastasis and 20.9% presented with distant metastasis. Initial tumor stages are shown in Table 1. In two patients the exact stage was not known due to undefined T-stage (both with local disease, N0M0). Four tumors were located above and 16 as invading or located below the superficial fascia. Satellite metastases were seen in two patients and a nodal skip-metastasis in one patient.

**Table 1:** Tumor staging at diagnosis (UICC/AJCC), summary of the R status of patients after primary surgery and final R status following all surgical treatments of primary disease at time of first diagnosis

Initial staging for all patients at diagnosis			
**Tumor size (initial T); median size 3.6cm (range, 0.3–11.1cm), all cases**			
T1 ≤ 5	n=29		
T2 > 5cm	n=8		
T3 > 10cm	n=1		
T4 > 15cm	n=0		
**Synchronous N and M (in first 3mos), all cases**			
N0M0	n=26		60.5% local
N1M0	n=8 (incl. one skip met.)		18.6% N1
NxM1	n=1		
N1M1	n=7		20.9% M1
N0M1	n=1		
**Stages UICC 8th edition (2017) (only CCS, n=40)**^**a**^ [9]			
Stage II	N0M0	T1	n=18
Stage IIIA	N0M0	T2	n=6
Stage IIIB	N0M0	T3	n=0
Stage IIIC	N1M0	T any	n=8
Stage IV	any N, M1	T any	n=7
**Stages AJCC** **8th** **edition: soft tissue sarcoma in the trunk and extremity, n=40 (2017)**^**a**^ [9]			
Stage II	N0M0	T1	n=18
Stage IIIA	N0M0	T2	n=6
Stage IIIB	N0M0	T3	n=0
Stage IV	N1 and/or M1	T any	n=15
**TNM AJCC 8th edition: abdomen and thoracic visceral organs, n=3 (stages not defined) (2017)**			
Tx	n=1, NxM1		
T3	n=2 (n=1, N0M0; n=1, N1M0)		
**R status after primary tumor resection (n=39 patients with resection)**			
**Cases of primary resection or resection biopsy**			
Resection biopsy	n=31	initial 81% R+	tumor size: ∅ 3.3cm (in median)
Biopsy (n=12) and resection	n=8	initial 12.5% R+	tumor size: ∅ 6cm (in median)
**R status after primary surgical treatment**			
R0	n=12	30.8% R− of all primary res.	
R1	n=14	64.1% R+ of all primary res.	
R2	n=7		
R1 or R2	n=4		
**Final R status**			
R0	n=28	71.8% R− of all final res.	
R1	n=9	25.6% R+ of all final res.	
R2	n=1		

*Met* metastasis; *Res* resection; *R*-: R0; R+: R1 or R2; *mos* months; *incl* including. ^a^does not include the cases in which the primary tumor arose from visceral organs or head and neck.

## Pathology

Of 26 patients with reported fusion analysis of our local database, 24 patients (92%) were shown to harbor an *EWSR1::ATF1* fusion. This chimeric translocation was seen in all GINET tumors except one case that carried a *EWSR1::CREB1* fusion detected by FISH analysis. All tumors presented *BRAF* wild type, except for one GINET. 90.6% of analyzed tissue was found to be positive for S-100 (incl. 2/3 GINET), 87% for HMB-45 (1/2 GINET) and 77.4% for Melan-A (1/3 GINET) staining. In nine further cases, we also found positive staining for SOX-10, in eight for Vimentin and in three for bcl-2. Tumor material was re-/assessed by reference pathologists with CCS/GINET confirmed in all cases.

## Initial treatment in locoregional disease

### Resection

Primary treatment was surgery in 39 patients (Table 1) and the vast majority received surgery outside of experienced centers (33/39, 84.6%). 69.2% of all patients with resection revealed no distant metastasis at diagnosis (N0M0, n = 26; N1M0, n = 8) and all patients with N0M0 status received a tumor excision. First surgery resulted in positive margins in 25/39 (64.1%) patients. However, in those patients with resection biopsy (n = 31, 79.5%), residual tumor was found in 25 of 31 patients (81%) (R1: 20, R2: 5). Of note, the median tumor size was 3.3 cm in patients with resection biopsy.

A *biopsy* was performed in 12 patients (median size at biopsy: 6 cm, all but one > 3 cm). In eight patients CCS was resected after biopsy, all with R0 status, except one with R1. In three cases resection was not performed due to multifocal progression at distant sites (M + :3) and in one case the resection status was unknown due to short follow-up (< 1mos, N + :1).

Sixteen patients with incomplete resection of local disease (of n = 26 in total N0M0 cohort, Table 1) were converted into R0 resections with 71.8% (n = 28) of all patients finally receiving R0 resections (Table 1). Two patients received an amputation of their lower leg, and one patient underwent exarticulation of the proximal phalanx. Additional local treatment to primary tumor resection was performed in 51% (n = 22).

### Lymphadenectomy and sentinel biopsy

Lymphadenectomy (8 of 11 positives for CCS) was typically performed in patients with visible lymph nodes in pre-surgical imaging studies of the adjacent region. A sentinel biopsy was done in five cases with two samples positive for CCS in immuno-histopathology. These two patients underwent subsequent lymphadenectomy with no evidence of further lymph node metastases in respective surgical specimen.

### Isolated limb perfusion

Twelve patients underwent isolated limb perfusion with TNF-α and melphalan, of which three cases were treated pre-operatively (all final R0) at small tumor sizes T1-T2 (N0M0 = 2: both with lymph node recurrence after 1.9 years and 6.5 years; time from diagnosis to progression 3.1 and 7 years), and in five cases as additive treatment following R + resection without an option for additional, limb-salvaging surgery (time to recurrence 0.4–1.1 years/ time to progression 0.5–2.5 years). Four other cases received ILP following local, symptomatic tumor recurrence. In all patients, radiologic, metabolic (FDG-PET) and clinical response was observed. However, all patients experienced tumor progression outside the treated region after 0.9–11.8 months). ILP was part of a multimodal treatment strategy including postinterventional resection, whenever possible.

### Radiotherapy

Thirteen patients were identified to have received local radiotherapy as initial therapy. Four of these were treated in a neoadjuvant (one at difficult anatomical location; two with lager tumor size to ensure limb salvage; one patient within palliative context: N1M1; all were followed by R0 resection) and two in an adjuvant setting after R0 resection with close surgical margins (up to 0.1 mm), six cases following positive microscopic margins (all R1) and one as single therapy without resection. The median radiation dose (incl. boost therapy) was 63.2 Gy (range: 50–70 Gy). Boost therapy ranged from 10 to 26 Gy. One patient with N1M0 at diagnosis received radiotherapy of the affected lymph pathways.

### Systemic therapy

Eleven patients were treated with chemotherapy as initial treatment. Six patients with localized disease (n = 1) or locoregional spread (N1: n = 5) received chemotherapy doxorubicin + ifosfamide, dacarbazine + ifosfamide or temozolomide. Two patients received neoadjuvant chemotherapy, one patient due to a large tumor size with 11.1 cm (one cycle of doxorubicin + ifosfamide) followed by tumor resection and the other patient in a palliative setting (with M1 at diagnosis).

### Patterns of recurrence

**Fig. 1:**
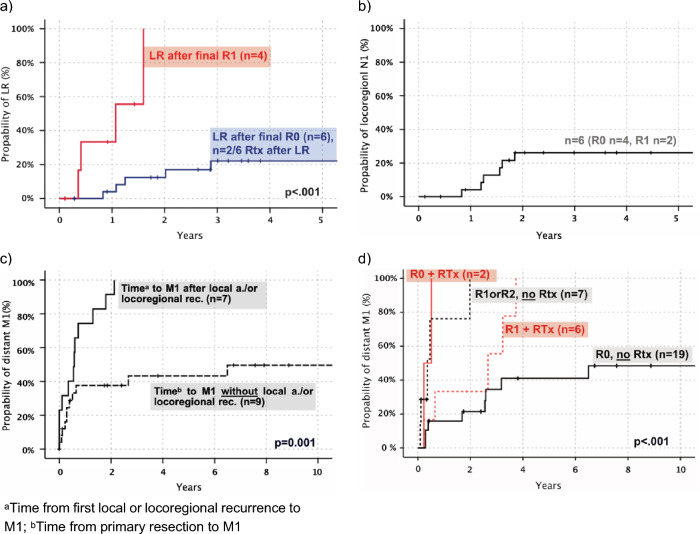
Patterns of local, locoregional (N1) and distant recurrence (M1) in CCS. **a** Local recurrence (LR, n = 10, only primary tumor location) in patients with final R0 vs final R1, including two patients with RTx in R0 cohort (time primary resection—recurrence, in years). **b** Locoregional (N1) recurrence after resection in patients with local disease, N0M0 (time primary resection—recurrence, in years). **c** Time to distant metastasis after local treatment (R0) for regional relapse (local or N1) compared to patients without regional relapse (time from resection to distant metastasis, in years). **d** Distant metastasis-free survival for patients with complete (R0) vs incomplete resection (R1 or R2) and postoperative radiotherapy (time primary resection—distant metastasis, in years)

In 18 of 24 patients with no distant metastases (N all, M0) at diagnosis, CCS recurred (at local site or distant) after a median time of 1.2 years – relapses did not occur beyond three years from time of primary resection with R0 resection status (Fig. 1a, b).

Five-year local recurrence-free survival for all patients was 59%. Patients with R0 resections presented a probability of 22% LR (local relapse) in three years (Fig. 1a). All locoregional lymph node recurrences developed in the first two years with a rate of 26% (R all, initial N0M0) (Fig. 1b). In patients with complete final resection, median time to local recurrence was 1.6 years (n = 6; range, 0.8–12.5yrs), compared to 0.7 years (n = 4; range, 0.4–1.6yrs) in patients with incomplete microscopic resection (only those with R1). Within two years local recurrence occurred in all patients with R1 but only 13% of patients with R0 resection (negative margins) (Fig. 1a).

In total, 19/28 (68%) of patients with complete resection (R0) showed distant metastasis after five years in Kaplan–Meier estimation. Patients with local recurrence were at a much higher risk of developing distant metastasis (all of them show M1 after two years, Fig. 1 c) with a median time to distant metastasis of 0.6 years vs 15.2 years. M1 occurred always after local or locoregional (N1) tumor re-appearance (only one patient with M1 before locoregional N + , ∆ 6.6mos). The primary tumor size did not correlate with distant metastasis (p = 0.475).

Patients with postoperative radiotherapy after R0 resection (n = 2, both at close primary resection) presented with distant metastasis in the first year after resection and patients with R0 and no radiotherapy (n = 19) presented with a median survival of 15.2 years (Fig. 1d). All patients with R1 and radiotherapy showed a metastatic-free survival of 66% vs 40% in the R1 group without radiotherapy at two years (and 0% with R2 included, Fig. 1d). In-field recurrence at local site after primary resection in patients with N0M0 (n = 8) was seen in 37.5% (n = 3) of patients, which included two with R0 (25% in-field) and one with R1 status (follow-up in-field recurrence, range: 0.2–2.7yrs).

In all patients the median time to distant metastasis was 1.1 years. 26% (n = 5) with CCS reappearance developed distant metastatic spread as their first tumor recurrence (median 2.7yrs). At first observation, 14 tumors had spread in only one remote organ: lung n = 5, bone n = 3, soft tissue n = 2, liver n = 1, indefinable n = 1 and in 14 patients, multiple organs were infiltrated. 65.5% of all metastases (n = 29) were thoracic (mostly pulmonary: n = 17, 58.6%), 44.8% (n = 13) were found in the abdominal cavity (mostly hepatic: 13.8%) and 20.7% (n = 6) showed involvement of the spinal system. Half of all pulmonary metastases were observed within the first year of diagnosis (median, 0.96yrs). Both the chest as well as abdominal cavity were infiltrated after 1.4 years in median. CCS progressed four months (in median) after tumor recurrence or five months after initial stage IV (N + and/or M + : 8th edition AJCC staging). 80% of patients with progressive disease died during follow-up. Time from disease progression to death was about half a year (median 6.4mos).

### Treatment of local (LR), locoregional recurrence (N1) and distant metastasis

All patients with local recurrence (in total n = 10 with LR) were successfully treated with surgery. In patients with LR two received radiotherapy. Notably, tumor recurrence after surgery emerged in locoregional lymph nodes in seven patients which were clinically diagnosed by ultrasound. All of them underwent lymph node dissection which revealed CCS-positive lymph nodes. Additionally, six patients with positive lymph node removals received radiotherapy of the corresponding region.

Distant metastases were treated with surgery in eight patients, and 21 patients with metastatic disease received chemotherapy in a palliative intention. Only one of those patients experienced tumor regression with doxorubicin + ifosfamide in combination with hyperthermia. Further treatments of metastatic CCS included checkpoint inhibitors such as ipilimumab, nivolumab and pembrolizumab, the kinase inhibitors crizotinib, pazopanib and sunitinib, as well as experimental treatments with treatment durations ranging from less than a month up to one year.

### Overall survival

**Fig. 2:**
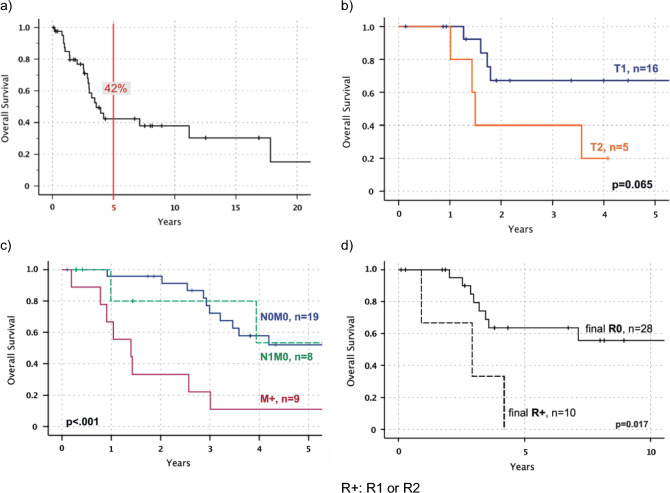
Overall survival (OS) depending on tumor size, staging at diagnosis and final R status in Kaplan–Meier estimation. **a** OS for all patients (time: diagnosis CCS—death, in years). **b** OS for all tumor sizes (T) only in patients with local disease (N0M0) and with final R0 (time: diagnosis CCS—death, in years). **c** OS depending on initial staging: local disease vs locoregional spread vs distant metastasis for all patients (time: diagnosis CCS—death, in years). **d** OS for final R0 vs final R + (R1 or R2) with localized disease at diagnosis in Kaplan-Meier estimation (N0M0) (time: diagnosis CCS – death, in years)

OS for all patients was 3.6 years in median with a 5-year survival rate of 42% (10-year survival 38%) (Fig. 2) and 52% for initially localized CCS (10-year survival 44%). All patients died of CCS or GINET. Only five patients with distant metastasis (n = 28) were alive at follow-up (median follow-up to survival 3.8yrs). Four patients survived more than ten years (follow-up: 12.5—26.9yrs). One of four patients was disease-free until distant M + showed up after 15 years (follow-up other three patients 12.5—26.9yrs). All of them were primarily localized and underwent complete resection (R0) as single therapy; only one received adjuvant ILP and CTx (regimens: ifosfamide, dacarbazine).

## Analysis of prognostic factors

### Univariate analysis

Notable, though not significant, was the 5-year survival difference between men and women with 39% vs 59% (p = 0.219, N0M0 all). No difference was found based on primary tumor location (Table 2). Tumor size (≤ 5 cm vs > 5 cm) showed no prognostic significance (p = 0.083, all N0M0) in univariate analysis (Table 2), but worth mentioning, patients with a tumor size of ≤ 5 cm in local disease lived up to 17.8 years (n = 19) in median vs 2.9 years and the metastasis-free survival was significantly longer (median, 3.8yrs vs 1.7yrs; p = 0.004). In patients with local disease at time of diagnosis (N0M0) and complete resection (final R0) smaller CCS with T1 presented a 2-yr survival 66% and T2 with 40% (p = 0.065, Fig. 2). T3 or higher was not represented.

**Table 2:** Univariate analysis of prognostic factors. a) Comparison of baseline characteristics and final resection status. b) Univariate analysis of N0M0 vs N1M0 vs M1M1 at diagnosis (left; all cases, n=43) and comparison of initial UICC and AJCC stages both 8th edition (right; only CCS, n=40) Left vs right column: ^a^p-value; ^b^5yr overall survival; ^c^median survival; N0M1 only n=1‚ thus excluded from statistical analysis

	All stages			Patients with local CCS (N0M0)		
	*p*-value	5 yr-OS	Median OS	*p*-value	5 yr-OS	Median OS
**Gender**: men (n = 18) vs women (n = 25)	0.111	31% vs 48%	3yrs vs 4.2yrs	0.219	39% vs 59%	3.6yrs vs 17.8yrs
**Tumor** **size**: > 3 cm (n = 23) vs ≤ 3 cm (n = 15)	0.118	27% vs 52%	2.9yrs vs 17.8yrs	0.363	44% vs 58%	3.5yrs vs 17.8yrs
**Tumor size**: > 5 cm (n = 9) vs ≤ 5 cm (n = 29)	0.087	23% vs 47%	2.9yrs vs 4.1yrs	0.083	33% vs 57%	2.9yrs vs 17.8yrs
**Location** **only on** **lower extr.**: proximal leg (above knee) (n = 5) vs foot (n = 12)	0.520	30% vs 42%	2.9yrs vs 4.1yrs	0.628	35% vs 57%	4.2yrs vs 7.1yrs
**Location on all extr.**: proximal (n = 6) vs distal site (incl. elbow or knee joints) (n = 29)	0.708	–	–	0.995	–	–
**Resection status**: final R+ (n = 10) vs final R0 (n = 28)	* < .001*	*0%* vs 60%	1.4yrs vs 17.8yrs	*0.017*	*0%* vs 64%	2.9yrs vs 17.8yrs

*R +*  R1 or R2; *OS* overall survival; *extr*. extremities; *incl*. including


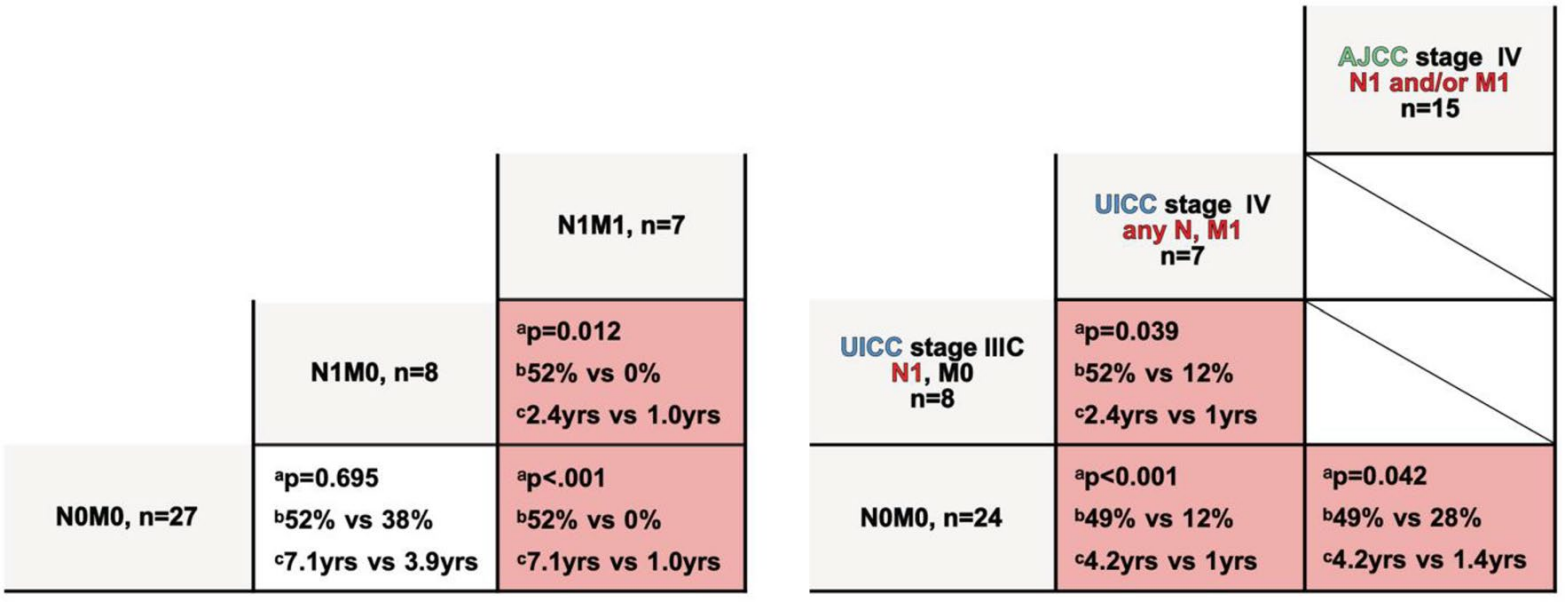


Figure 2 shows stage specific survival of local N0M0, N1M0 and distant disease at diagnosis. Patients with metastatic disease revealed a survival of one year vs 7.1 years in N0M0. A univariate analysis of NM and comparison of UICC/AJCC staging for soft tissue sarcoma is shown in Table 2.

Complete resection as final surgical outcome (n = 28) correlated significantly (p = 0.017) with longer survival rates compared to R + status (n = 10) in localized CCS with a difference in 5-year survival of 0% vs 64% and 2.9 years vs 17.8 years in median (Fig. 2, Table 2).

### Multivariate analysis

Multivariate analysis applying Cox regression with fixed covariates showed that wide resection margins (R0) were associated with a superior survival (p = 0.019, HR for R + : 6.7, CI: 1.3–33-3). Initial M + presented a 11.5 times higher HR (p = 0.048, 95% CI: 1–122.6). Sex was as a significant predictor (p = 0.021) with a higher risk for men (HR 5.5, 95% CI: 1.3–23.6). Included predictor variables without significant impact were age (> 40yrs vs ≤ 40yrs), tumor location (elbow/knee and distal vs proximal extremity) and tumor size (T1 vs > T1).

## Discussion

Clear cell sarcomas represent a sarcoma subtype notoriously known for a high rate of metastatic spread and a dismal prognosis in patients with metastatic disease, as currently no active medical treatment is available. We here present one of the largest single center analyses in this rare sarcoma subtype.

In line with other series, CCS were mainly located within the extremities with a median tumor size of 3.6 cm at diagnosis (Deenik et al. 1999; Nakai et al. 2020; Chung and Enzinger 1983; Kawai et al. 2007; Montgomery et al. 1993) (see Table 3). The superficial location may explain the relatively low tumor size at diagnosis compared to other sarcoma. However, the outcome in our series was rather poor (42% 5 yr-OS) compared to other series (Table 3). This may be explained by the exceptionally high rate of synchronous metastases (N1: 18.6%, M1: 20.9%) in our cohort.

**Table 3:** Overview and comparison of previous studies

Year	Previous studies	No. of pat	Median size	5 yr-OS, all pat.	Lymph node involvement N1M0 at diagnosis (a. survival)	Distant metastasis at diagnosis (a. survival)
1965	Enzinger (1965)	21	4 cm	n.a	n.a	n.a
1983	Chung & Enzinger (1983)	141	3.3 cm	n.a	n.a	n.a
1990	Sara et al. (1990)	17	4.5 cm	40%	1 pat	1 pat
1992	Lucas et al. (1992)	35	4.5 cm	67%	11.4%	n.a
1993	Montgomery et al. (1993)	58	2.5 cm	63%	n.a	n.a
1999	Deenik et al. (1999)	30	3 cm	54%	10%	10%
2001	Finley et al. (2001)	8	5 cm	55%	n.a	n.a
2002	Ferrari et al. (2002)	28	4 cm	66% (63.3%, 5 yr-EFS)	17.9% (60%, 5 yr-EFS)	7.1%
2007	Kawai et al. (2007)	75	4 cm	47%	16%	14.7%
2008	Clark et al. (2008)	35	4 cm	52%	n.a	n.a
2012	Hocar et al. (2012)	52	4.8 cm	69%	3.8%	1 pat
2014	Bianchi et al. (2014)	31	3 cm	72%	19% (40%, 2 yr a. 0%, 5 yr-OS)	6.5% (0% 2 yr, a. 0%, 5 yr-OS)
2018	Gonzaga et al. (2018)	489	4.2 cm	50%	6%	15%
2022	Smrke et al. (2022)	55	n.a	≈10% (median OS 15 mos)	24%, N1 and/or M1	
2024	Cohort reported herein	**43**	**3.6 cm**	**42%**	**18.6% (52%, 5 yr-OS)**	**20.9% (16%, 5 yr-OS)**

*OS* overall survival; *EFS* event-free survival; *no.* number; *pat.* patients; *a.* and; *n.a.* data not available

In our study, the multivariate analysis but not the univariate analyses revealed male gender to be associated with a poorer overall survival (p = 0.021, HR: 5.5) which is in line with a study by Kawai et al. (2007). The distribution of tumor size and final resection status was similar in both groups. Of note, tumors ≤ 5 cm in size were significantly less likely to develop metastases (p = 0.004) and showed a trend towards a better median overall survival rate (17.8yrs vs 2.9yrs, p = 0.067) in patients with R0 resection (Table 2). Notably, even patients with a smaller tumor size (T1) had a tumor-related mortality of 33% within two years (after final R0). Both, Sara et al. (1990) and Bianchi et al. (2014) found a tumor size of more than 5 cm to be associated with a poor survival (≤ 5 cm vs > 5 cm: 83% vs 25%, 5 yr disease specific survival (Bianchi et al. 2014). We conclude that a tumor size that dichotomizes between ≤ 5 and > 5 cm could be helpful in a prognostic system.

### Local therapy

The resection of clear cell sarcoma should be planned in advance. This means, that a pre-resection biopsy should be perfomed if CCS is suspected to prevent “whoops” procedures. In this cohort, planned resections following tumor biopsies led to a dramatically lower number of R + resections compared to resection biopsies for suspected benign lesions (12.5% vs 81% R1/R2, respectively). European guidelines recommend resection biopsies for tumors below 3 cm (Casali et al. 2018; Dangoor et al. 2016). However, these would spare structures as tendons or joints which are commonly infiltrated by CCS. In case of positive surgical margins in locoregional disease (N0M0, N1M0) a re-resection should be aimed for (Fig. 2). Patients with positive margins and R0 re-resection had a better survival compared to R + resections. Incomplete (R1) surgery was associated with a local recurrence rate of 100% within two years and the median time from LR to death was only 2.8 years.

Sentinel biopsies revealed positive lymph nodes in two of seven patients without evidence of distant metastases. Notably, in these two patients lymphadenectomy (LAD) did not reveal further metastases and patients were disease free at two and 7.5 years follow-up. Our data does not allow any conclusions but provides evidence that even in patients without apparent lymph node involvement, sentinel biopsies may uncover metastatic disease. More data is needed to further determine the role of both biopsies as well as of LAD in general (Al-Refaie et al. 2004).

Hocar et al. (2012) observed improved survival rates in patients with wide resection (R0). In our series, only 33.3% of all patients who were resected for localized disease (n = 9) achieved a R0 status and even in those patients, local and locoregional recurrence rates were high (LR: 22% after two years; locoregional relapse: 26% after two years). Survival rates of R0-resected patients were similar compared to cohorts published within the last 30 years. This underscores the lack of effective adjuvant treatment options (Kawai et al. 2007; Hocar et al. 2012; Nakai et al. 2020).

We hypothesize that correct diagnosis, staging and concomitant wide resection as primary treatment would improve overall survival – particularly as radiation was not able to salvage poor surgery in this cohort. Most notably we observed in-field recurrences in two patients providing additional evidence that CCS may be much less sensitive to irradiation than most other sarcomas. Determining the therapeutic role of additive radiotherapy and multimodal treatments particularly after incomplete resection (R1 or R2) remains a challenge in clinical practice.

Isolated limb perfusion is a therapeutic approach frequently used in patients with advanced sarcomas that may otherwise be candidates for amputation. Some patients with CCS derived clinical benefit from ILP (Grabellus et al. 2011). Given the obvious lymphatic spread of clear cell sarcomas ILP has served as a rationale at our center for its use. Our small series reported herein precludes a general recommendation for ILP and a prospective trial may be needed to determine clinical benefit (beyond palliative relief).

### Metastatic disease and systemic therapy

Two staging systems are frequently used for patients with soft tissue sarcomas. The UICC and AJCC (for trunk and extremity) classifications (both 8th edition of 2017) are highly similar except for UICC separating N1 as non-metastatic compared to the AJCC system which defines N1 as stage IV.

In our cohort, the presence of N1M0 did *not* show a significant difference in survival when compared to local disease (N0M0) in multivariate analysis including the resection status as predictor variable (p = 0.541, HR: 0.5). 52% of patients with locoregional spread (N1, M0) were alive after five years of diagnosis and only 15% with distant metastasis (M1) at diagnosis (Fig. 2). A study by Behranwala et al. (2004) supports these findings (n = 73, 1y-OS N1M0 77.5% vs. NxM1 36.3%, p = 0.005).

**Fig. 3:**
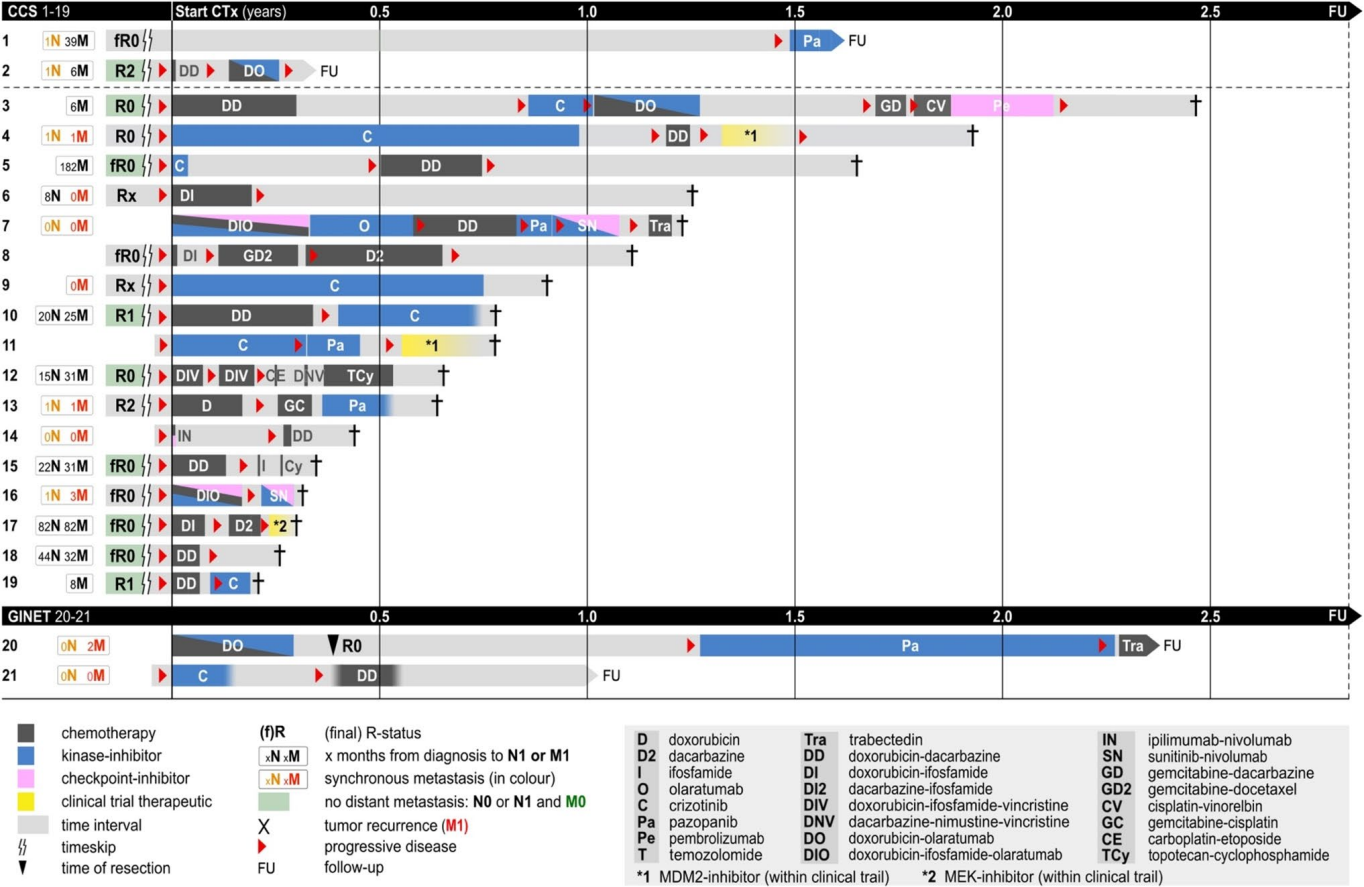
Application of different chemo- and targeted therapies in patients with metastatic (N any, M1) CCS or GINET

At our center patients with metastatic disease were treated with a multitude of systemic therapies (Fig. 3). Most patients experienced an immediate progression upon treatment with chemotherapy, kinase inhibitors or immune-checkpoint inhibitors. Not a single objective remission was observed. This is supported by A. Constantinidou et al. (2010) reporting a 4% response rate for chemotherapy. While Negri et al. proposed the use of sunitinib (Negri et al. 2012) a multi-tyrosine kinase inhibitor (TKI) or pazopanib, a VEGFR-inhibitor with potential effects on hepatocyte growth factor receptor (MET) (Outani et al. 2014), in our study group all patients immediately progressed on both drugs (Fig. 3). However, two patients treated with crizotinib, a multi-TKI targeting MET, were still stable for at least three months in our cohort (Fig. 3, pat. 9 and 10) but this could also be due to a more indolent course of their disease. Reported series did also include GINETs (A. Smrke et al. 2022). However, differences in drug sensitivity of CCS and GINETs were not evaluated or not reported (median survival CCS-GIT 13.5 months and for GNET 9.5 months). Given the low numbers in our series, we cannot make further conclusions, even though both GINET patients were alive at the time of last follow-up. We emphasize multicentric retrospective analyses to further address this question.

Taken together, we believe patients should be informed about the very limited value of currently available systemic treatments. Clinical trials or best supportive care should represent the standard of care. However, the addition of locoregional and systemic treatments even within the metastatic setting might prolong individual patient’s survival.

## Conclusion

This study provides further evidence regarding prognosis, risk factors and therapeutic options in the treatment of CCS and GINET to improve patient consultation. This includes our own cohort as well as data from previous publications. Often misdiagnosed as benign lesions, we found that initial biopsy and adequate pathological diagnostics including fusion analysis might prevent from inadequate surgical removal. In contrast to other sarcoma lymphonodular spread was common and prognostically relevant. Hence, initial staging including functional imaging and consecutive sentinel lymph node biopsy is crucial for further treatment planning and patient consultation. In contrast, Bianchi et al. (2014) found that locoregional spread had a similar prognosis as distant metastasis at diagnosis. However, hardly any study addresses survival in patients with initial lymph node involvement (Table 3; Ferrari et al. 2002; Gonzaga et al. 2018; Bianchi et al. 2014). Additional risk factors were male gender and a tumor size > 5 cm. We identified complete surgical removal (primary or secondary R0) as the most important treatment, whereas the success of radiotherapy and systemic treatment was very limited. Isolated limb perfusion might be discussed in patients with skip lesions or lymphonodular spread at diagnosis. Crizotinib was the only systemic treatment option that led to short period of disease stabilization of three months in two patients within the metastatic setting. However, targetable genetic alterations have not been identified in this disease. As data of prospective clinical trials for CCS treatment is lacking, there is an unmet need to generate evidence for locoregional and systemic, targeted treatment options in this ultra-rare disease.

## Supplementary Information

Below is the link to the electronic supplementary material.

Supplementary file1 (DOCX 15 KB)

## Data Availability

All other authors have no relevant financial or non-financial interests to disclose. No datasets were generated or analysed during the current study.
