# Supplementary material for: Prognostic factors in clear cell sarcoma: an analysis of soft tissue sarcoma in 43 cases
Source: J Cancer Res Clin Oncol. 2024 Nov 13;150(11):494. doi: 10.1007/s00432-024-05980-3 (PMC11560989; doi:10.1007/s00432-024-05980-3)
Supplement: Supplementary file 1 — Supplementary file1 (DOCX 15 KB) [file 432_2024_5980_MOESM1_ESM.docx]

**Supplementary Table 2**

Multivariate analysis (Cox regressionᶠ)

| **Factors**ᶜ | | **p-value** | **HR**ᵈ | **95% CI**ᵉ | |
| --- | --- | --- | --- | --- | --- |
| age: **>40** vs <=40 yrs | | 0.243 | 0.38 | 0.1 - 1.9 | |
| sex: **men** vs women | | **0.021** | **5.5** | 1.3 - 23.6 | |
| location extremity: **distal**ᵃ vs proximal | | 0.182 | 3.2 | 0.6 - 18.8 | |
| size: **>5cm** vs ≤ 5cm | | 0.180 | 4.1 | 0.5 - 18.8 | |
| stage: | **N0M0** | 0.121 | - | - | |
|  | **N1M0** | 0.541 | 0.5 | 0.1 - 4.4 | |
|  | **N0M1** | **0.048** | **11.2** | 1.0 - 122.6 | |
| resection status: **fR+** vs fR0ᵇ | | **0.019** | **6.7** | 1.4 - 33.3 | |
| ᵃelbow or knee and distal; ᵇfinal rescetion status; ᶜall factors included in Cox analysis had propotional Hazards; ᵈHR=hazard ratio; ᵉ95% HR confidence intervall; ᶠOmnibus-test = p<.001 | | | | |  |
